# Association between Sleep Habits and Metabolically Healthy Obesity in Adults: A Cross-Sectional Study

**DOI:** 10.1155/2017/5272984

**Published:** 2017-03-06

**Authors:** Thirumagal Kanagasabai, Ramandeep Dhanoa, Jennifer L. Kuk, Chris I. Ardern

**Affiliations:** School of Kinesiology and Health Science, York University, Toronto, ON, Canada

## Abstract

Higher body mass index (BMI) increases the risk of cardiometabolic diseases, but nearly a third of the people living with obesity (BMI: ≥30 kg/m^2^) are metabolically healthy (MHO). Extreme sleep durations and poor sleep quality are associated with higher bodyweight and cardiometabolic dysfunction, but the full extent to which sleep habits may help differentiate those with MHO versus metabolically abnormal obesity (MAO) is not yet known. Data from the U.S. National Health and Nutritional Examination Survey 2005–08 was used (BMI: ≥30 kg/m^2^; ≥20 y; *N* = 1,777). The absence of metabolic syndrome was used to define MHO. Those with MHO tended to be younger, female, Non-Hispanic Black, never smokers, more physically active, and with less physician diagnosed sleep disorders than MAO. Neither sleep duration nor overall sleep quality was related to MHO in crude or multivariable adjusted analyses; however, reporting “almost always” to having trouble falling asleep (OR (95% CI): 0.40 (0.20–0.78)), waking up during the night (0.38 (0.17–0.85)), feeling unrested during the day (0.35 (0.18–0.70)), and feeling overly sleepy during the day (0.35 (0.17–0.75)) was related to lower odds of MHO. Selected sleep quality factors, but not sleep quantity or overall sleep quality, are associated with the MHO phenotype.

## 1. Introduction

Despite the well-known complications of excess body fat [1, 2] emerging research suggests that up to 30% of people living with obesity (body mass index of ≥30 kg/m^2^) are metabolically healthy (MHO) [3]. MHO individuals may also be insulin-sensitive [4] and have a similar mortality risk as metabolically healthy normal bodyweight individuals [5, 6]. However, the existence of the MHO phenotype as a distinct entity, particularly over time, remains controversial [7]. Indeed, a recent meta-analysis suggests that, with longer follow-up, MHO individuals are at a 24% greater risk of cardiovascular events and all-cause mortality compared to the metabolically healthy normal-weight people whereas metabolically* unhealthy* individuals are at similarly elevated morbidity and mortality risks regardless of their body weight class [8]. Therefore, identifying factors that help maintain the metabolic health of individuals living with higher bodyweight may help reduce their morbidity and mortality risks.

Unique determinants that differentiate MHO and metabolically abnormal obesity (MAO) are largely unknown. However, lifestyle factors such as physical activity, weight history [9–11], dietary habits [3, 12, 13], and systematic inflammation and location of fats storage [14] have been found to differ between MHO and MAO. Sleep is another potential behavior that can vary between MHO and MAO phenotypes; however, in the only two studies conducted thus far, one found no difference in self-reported sleep duration between MHO and MAO [15], and the other found higher sleep duration in MHO women (MHO: 7.6 h versus MAO: 7.0 h), but not men [3]. Neither study [3, 15] evaluated the relationship between sleep quality alongside sleep duration and MHO. They either focused on middle-aged adults [3] or were limited by the application of ethnic-specific waist circumference cut-offs to a Korean sample [15]. To date, the relationship between sleep habits and the MHO phenotype amongst the U.S. adult population has yet to be studied. The primary purpose of this study was to, therefore, estimate the relationship between sleep duration and quality factors of MHO versus MAO in free-living U.S. adults.

## 2. Subjects and Methods

### 2.1. Sample

Data for this analysis was derived from the U.S. National Health and Nutrition Examination Survey (NHANES). Briefly, NHANES is a series of cross-sectional studies designed to assess the health and nutritional status of the U.S. population and includes demographic, socioeconomic, dietary, and health-related information in the interview component and medical, dental, and physiological information in the physical examination component [16]. Approximately 5,000 individuals are sampled through a complex probability cluster design on an annual basis. As a result, the NHANES 2005–2008 cycles had an initial sample of 20,497 people. Exclusions were made in sequence for age (<20 y: *n* = 9,583), BMI (<30 kg/m^2^: *n* = 13,499), pregnancy (*n* = 136), missing metabolic syndrome (MetS) components or implausible values (*n*_triglycerides_ = 241, *n*_blood  pressure_ = 95, *n*_waist  circumference_ = 177, *n*_HDL  cholesterol_ = 91, and *n*_fasting  plasma  glucose_ = 1,686), and sleep duration (*n* = 7), leaving a final analytic sample of 1,777.

### 2.2. Metabolically Healthy Obesity and Metabolically Abnormal Obesity

The Joint Interim Statement for MetS was used as the indicator of cardiometabolic health in those living with obesity based on the BMI cut-off of ≥30 kg/m^2^ [17]. That is, those with ≤2 MetS components were classified as MHO: elevated waist circumference: men: ≥102 cm, women: ≥88 cm; elevated fasting triglycerides (≥1.69 mM) or medication; low HDL cholesterol: men: <1.04 mM, women: <1.29 mM, or medication; elevated systolic (≥130 mmHg) and/or diastolic (≥85 mmHg) blood pressure or medication; and elevated fasting plasma glucose (≥5.6 mM) or medication [10, 17]. Those with ≥3 of the above measures were classified as MAO [17].

### 2.3. Sleep Habits

NHANES administered the Sleep Disorders Questionnaire to individuals aged ≥16 y to assess typical sleep habits for the past month [16]. The Sleep Disorders Questionnaire contains items from two previously validated sleep questionnaires [18]. Information on the typical sleep duration (hours (h) per night) on weekdays or workdays was collected as whole numbers [1 to 11 and ≥12 h] from a single question [“How much sleep do you usually get at night on weekdays or workdays?”] [16]. The responses were categorized as ≤4, 5-6, 7-8, and ≥9 h per night [19, 20]. Overall sleep quality was determined from six questions that were used to obtain information on the sleeping habits of the participants in the past month: “How often did you have trouble falling asleep?”; “How often did you wake up during the night and had trouble getting back to sleep?”; “How often did you wake up too early in the morning and were unable to get back to sleep?”; “How often did you feel unrested during the day, no matter how many hours of sleep you have had?”; “How often did you feel excessively or overly sleepy during the day?”; and “How often did you not get enough sleep?” Responses to each question [0 = never; 1 = rarely (1 time a month); 2 = sometimes (2–4 times a month); 3 = often (5–15 times a month); and 4 = almost always (16–30 times a month)] were summed to obtain the overall sleep quality score, which was then categorized as good (0 to <3); fair (3 to <7); poor (7 to <12); and very poor (≥12 to 24) [21, 22].

### 2.4. Covariates

Age (20 to <40 y, 40 to <65 y, and ≥65 y), sex, ethnicity (Non-Hispanic White, Non-Hispanic Black, Mexican American, and Other), annual household income, education, alcohol intake, smoking history, leisure-time physical activity, and physician diagnosed sleep disorder were considered as covariates [10, 23]. Smoking was categorized as current, past (if smoked ≥100 cigarettes in one's life but not a current smoker), or never (if smoked <100 cigarettes in one's life) [10]. Educational attainment was categorized as <high school, high school, and college; income as <$20,000, $20,000–44,999, and ≥$45,000; and alcohol intake as 0, <3, and ≥3 drinks per day [23]. For leisure-time physical activity (PA), the metabolic equivalent (MET) scores provided by NHANES were used to calculate MET min/week, which were then categorized as no reported PA data, low PA (1 to 499 MET min/week), and guideline adherence (≥500 MET min/week) [10, 24]. Physician diagnosed sleep disorders were obtained from a single question [“Have you ever been told by a doctor or other health professional that you have a sleep disorder?”] [16].

### 2.5. Statistics

Mean and 95% confidence interval (CI) for continuous variables and percentage and 95% CI for categorical variables were determined for MHO versus MAO. Differences in demographic and behavioral characteristics were assessed with independent *t*-test and *χ*^2^ tests, as appropriate. The mean sleep duration and overall sleep quality score were compared by sex and MHO status. Logistic regression was subsequently used to estimate the odds ratio (OR, 95% CI) for MHO. The crude OR of MHO (OR_c_), odds of MHO adjusting for age, sex, ethnicity, education, income, smoking, alcohol, and leisure-time physical activity (OR_adj_), and the odds of MHO additionally adjusting for physician diagnosed sleep disorder (OR_adj2_) were estimated. The moderating effects of age, sex, and physician diagnosed sleep disorder on the overall relationship were also explored. All analyses were weighted with the medical exam sample weights using SAS v9.3 (Cary, NC, U.S.). Statistical significance was set at *α* of 0.05.

## 3. Results

Table 1 shows the sample characteristics, grouped by metabolic health status. Overall, 28.3% of the adults living with obesity had MHO. MHO individuals tended to be more active, younger, female, and never smokers and were also less likely to be have a physician diagnosed sleep disorder or belong to the Non-Hispanic White ethnic group (*p* < 0.05). MHO and MAO individuals did not differ in education (*p* = 0.05), income (*p* = 0.16), or alcohol intake (*p* = 0.05). The mean (95% CI) sleep duration between MHO and MAO was also not significantly different (MHO: 6.63 (6.47, 6.78) versus MAO: 6.70 (6.62, 6.78) hours); however, MHO had a slightly lower overall sleep quality score (8.34 (7.84, 8.83)) than MAO individuals (9.11 (8.69, 9.53)).

**Figure 1:**
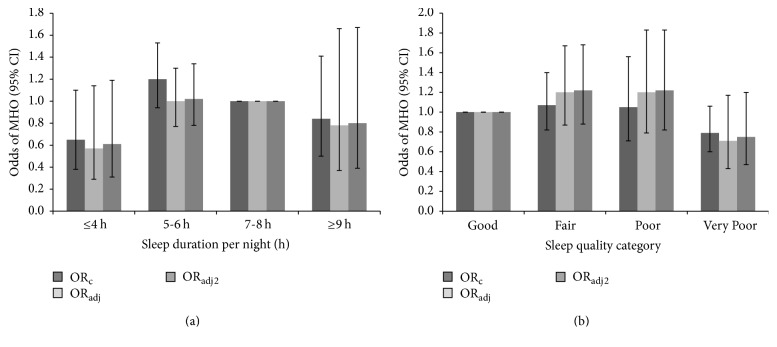
The odds of metabolically healthy obesity by sleep duration (a) and sleep quality (b) categories. Modeled for MHO (i.e., absence of metabolic syndrome according to the Joint Interim Statement, ≤2 cardiometabolic dysfunctions). Responses to sleep quality habit questions were summed to obtain the overall sleep quality, which were categorized as good (0 to <3); fair (3 to <7); poor (7 to <12); and very poor (≥12 to 24). OR_c_ is crude, OR_adj_ adjusted for age, sex, ethnicity, education, income, smoking, alcohol, and leisure-time physical activity, and OR_adj2_ additionally adjusted for physician diagnosed sleep disorder. MHO is metabolically healthy obesity in people with BMI of ≥30 kg/m^2^. All models were not significant.

**Figure 2:**
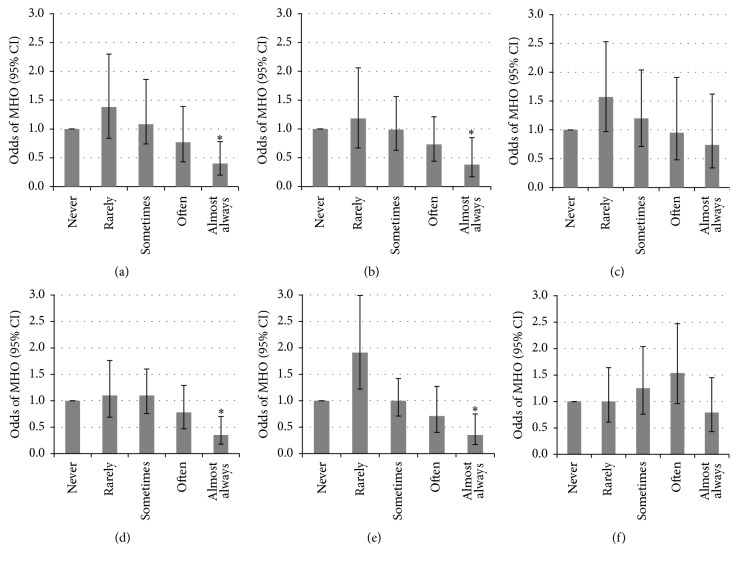
The odds of metabolically healthy obesity for each sleep quality question. (a) “How often did you have trouble falling asleep?”; (b) “How often did you wake up during the night and had trouble getting back to sleep?”; (c) “How often did you wake up too early in the morning and were unable to get back to sleep?”; (d) “How often did you feel unrested during the day, no matter how many hours of sleep you have had?”; (e) “How often did you feel excessively or overly sleepy during the day?”; and (f) “How often did you not get enough sleep?” Modeled for MHO (i.e., absence of metabolic syndrome according to the Joint Interim Statement, ≤2 cardiometabolic dysfunctions) adjusting for age, sex, ethnicity, education, income, smoking, alcohol, leisure-time physical activity, and physician diagnosed sleep disorder. Responses for each question were as follows: never; rarely (1 time a month); sometimes (2–4 times a month); often (5–15 times a month); and 4, almost always (16–30 times a month). MHO is metabolically healthy obesity in people with BMI of ≥30 kg/m^2^. ^*∗*^Significant difference.

In crude and fully adjusted models, sleep duration and overall sleep quality were not related to MHO (Figure 1), and no significant interactions (e.g., age, sex, and physician diagnosed sleep disorder) were found (all *p* > 0.05). When individual sleep quality questions were assessed, a significant relationship was found for four of the six questions, wherein those who reported “almost always” (versus “never”) had lower odds of MHO (e.g., “How often did you have trouble falling asleep?”; “How often did you wake up during the night and had trouble getting back to sleep?”; “How often did you feel unrested during the day, no matter how many hours of sleep you have had?”; and “How often did you feel excessively or overly sleepy during the day?”) (Figure 2). Only two sleep quality questions were not associated with MHO (i.e., “How often did you wake up too early in the morning and were unable to get back to sleep?” and “How often did you not get enough sleep?”). However, no significant relationships between the sleep parameters and the individual cardiometabolic components were found (data not shown).

## 4. Discussion

To our knowledge, this is the first population-based analysis to examine both sleep duration and sleep quality factors as they relate to MHO in free-living adults. Descriptively, MHO individuals reported slightly better overall sleep quality, but neither sleep duration nor overall sleep quality was associated with the odds of MHO in fully adjusted models. Nonetheless, regularly waking up during the night, feeling unrested during the day, feeling overly sleepy during the day, and trouble falling asleep were associated with lower odds of MHO. Therefore, our study provides novel evidence that specific sleep quality measures, but not sleep duration, are associated with the cardiometabolic health of people living with obesity.

### 4.1. Sleep Duration

Of the two previous studies that characterized the MHO versus MAO phenotype by sleep duration, one found a difference in women only [3], while the other supports our finding of no association [15]. In the study by Hankinson et al. [3] MHO women slept 7.6 h per day while MAO women slept 7.0 h. This is in contrast to our finding of no difference and may be attributed in part to Hankinson et al.'s [3] more conservative classification of MHO as the complete absence of diagnosed cardiometabolic abnormalities (versus the absence of preclinical Joint Interim Statement diagnostic cut-offs) [17] and their use of a slightly older sample. Although MHO tends to be younger than MAO and we did not find age was a moderator, the intersection of sleep and age requires further study [25], as both sleep duration and quality decrease [26], while cardiometabolic risk increases with age [27].

Further, chronic sleep deprivation promotes weight gain by altering specific endocrine pathways, such as leptin and ghrelin, that control appetite [28]. Sleep loss is also associated with higher preference for foods rich in fats and carbohydrates that promote weight gain [29]. Elevated sympathetic nervous system activity and higher inflammation have also been linked with poor sleep and obesity [30]. However, in the MHO phenotype, these potential mechanisms may remain intact while they may be compromised in the MAO phenotype [31]. Additionally, experimental evidence suggests that sleep loss results in metabolic consequences, including reduced nocturnal utilization of glucose that can promote insulin resistance and elevate diabetes risk in otherwise healthy individuals [28, 32]. A disproportionate number of individuals with diagnosed sleep disorders also have cardiometabolic dysfunction and obesity [33]. Therefore, although adequate sleep may be one of the common features that differentiate the MHO phenotype from the MAO phenotype, because of the complex interaction between sleep, obesity, and cardiometabolic dysfunction, the directionality of this relationship is not yet clear.

### 4.2. Sleep Quality

Results of this study suggest that select sleep quality measures, but not overall sleep quality, are associated with cardiometabolic health. These findings are supported by findings that poor sleep quality factors are related to obesity [34, 35], blood pressure [36], and glucose dysregulation [37, 38]. Indeed, individuals with obesity tend to have lower sleep quality and are more likely to experience a sleep disorder [39]. Mechanical [40] and physiological mechanisms [41] related to excess bodyweight could account in part for this decrease in sleep quality; however, whether obesity/higher bodyweight causes the decrease in sleep quality or vice versa remains unclear [30].

It remains to be seen whether overall sleep quality or efficiency, waking up during the night, daytime sleepiness, trouble falling asleep, sleep latency, or a combination of these factors is essential for cardiometabolic health and is an area in need of further study [21, 42]. Nonetheless, our study provides a new perspective on the complications of individuals with obesity in that MHO individuals have slightly better overall sleep quality than those with MAO, and selected sleep quality factors help predict the MHO phenotype. In our study, regularly reporting waking up during the night (i.e., 16–30 times a month), feeling unrested during the day, feeling overly sleepy during the day, and trouble falling asleep were moderately associated with the MHO phenotype [43]. However, some chronic conditions, for example, diabetes, hypertension, obesity (the clinical diagnosis), and depression, are also associated with fragmented sleep, insomnia, and excessive daytime sleepiness [44, 45]. Therefore, further research in this area is needed to better understand the relationship between various sleep quality factors and cardiometabolic health amongst those living with obesity/higher bodyweight.

### 4.3. Limitations

Several limitations exist with our study. First, due to the cross-sectional nature of our study, we cannot infer a causal relationship between sleep habits and MHO. Second, we were unable to assess the relationship between changing sleep patterns and napping within a 24 h period, which may give a better depiction of the sleep-MHO relationship. It is also possible that some yet to be assessed combination of sleep quantity and quality is a better predictor of MHO. Other cardiometabolic risk factors, such as percent body fat, HOMA-IR, and LDL-cholesterol, may also be indicators of the MHO phenotype but they were not included in our definition. Our definition for MHO compares favorably with much of the current literature, but MHO has been defined with many different definitions [46]. Further, we used self-reported sleep habits, which were susceptible to recall and healthy responder biases. Finally, we did not consider any other diseases (e.g., depression, coronary heart disease) or circumstances, such as shift-work, that affect sleep and circadian rhythm [47, 48]. Given the limitations of this study and current knowledge on the relationship between sleep and the MHO phenotype, research using objective measures of sleep within a longitudinal framework is needed. Particularly, studies to assess the importance of changing sleep patterns as they relate to the MHO versus MAO phenotypes are needed. Future research in this area should also quantify the contributions of other diseases and circumstances (mentioned above) to the sleep-MHO relationship.

## 5. Conclusions

Experiencing regular disturbances in sleep quality, but not overall sleep quality or sleep duration, is associated with MHO. Further studies in other populations living with obesity (i.e., higher bodyweight) are needed to confirm our findings.

## Figures and Tables

**Table 1:** Characteristics of the sample adult (≥20 y, obesity: BMI ≥ 30 kg/m^2^) population.

Characteristics	MHO	MAO	*p* value
	(*n* = 484)	(*n* = 1,293)	
Age (mean (95% CI))	42.3 (40.3, 44.4)	51.0 (50.7, 53.3)	<0.05
Age (% (95% CI))			
≥20 to <40 y	49.8 (43.9, 55.7)	20.7 (17.1, 24.4)	<0.05
≥40 to <65 y	40.9 (35.6, 46.2)	58.9 (55.4, 62.5)	
≥65 y	9.3 (6.0, 12.6)	20.3 (17.3, 23.4)	
Sex			
Males	39.2 (32.8, 45.7)	49.0 (45.7, 52.3)	<0.05
Females	60.8 (54.3, 67.2)	51.0 (47.7, 54.3)	
Ethnic			
Non-Hispanic White	58.6 (49.0, 68.3)	71.8 (66.4, 77.2)	<0.05
Non-Hispanic Black	21.5 (14.4, 28.6)	13.0 (9.5, 16.5)	
Mexican American	9.7 (5.3, 14.2)	7.1 (4.8, 9.5)	
Other	10.1 (6.3, 14.0)	8.1 (5.3, 10.6)	
Education			
<High school	19.1 (15.5, 22.6)	22.2 (18.8, 25.6)	NS
High school	22.1 (16.3, 28.0)	27.6 (24.1, 31.1)	
College	58.8 (53.6, 64.0)	50.2 (45.1, 55.3)	
Income			
<$20,000	14.4 (10.9, 17.9)	18.7 (15.7, 21.7)	NS
$20,000–44,999	30.8 (25.3, 36.2)	29.9 (25.5, 34.3)	
≥$45,000	54.9 (49.0, 60.7)	51.4 (46.4, 56.4)	
Smoking			
Never	58.9 (53.3, 64.5)	49.2 (45.7, 52.8)	<0.05
Current Smoker	17.1 (13.0, 21.3)	19.9 (16.7, 23.0)	
Past smoker	23.9 (20.0, 27.9)	30.9 (27.7, 34.1)	
Alcohol			
0 drinks/day	31.7 (26.0, 37.3)	38.7 (35.1, 42.3)	NS
<3 drinks/day	45.1 (39.4, 50.7)	42.2 (38.9, 45.5)	
≥3 drinks/day	23.2 (18.8, 27.7)	19.1 (16.5, 21.7)	
Leisure-time physical activity			
No reported PA	64.6 (57.7, 71.5)	70.1 (65.3, 75.0)	<0.05
1–499 MET min/w	10.5 (7.6, 13.3)	12.1 (9.3, 14.8)	
≥500 MET min/w	24.9 (20.1, 29.8)	17.8 (14.1, 21.5)	
Sleep hour			
≤4 h	4.6 (2.8, 6.3)	7.3 (5.4, 9.2)	NS
5-6 h	39.6 (34.7, 44.5)	34.1 (30.5, 37.7)	
7-8 h	50.9 (45.8, 56.0)	52.5 (48.9, 56.1)	
≥9 h	4.9 (2.6, 7.3)	6.1 (4.7, 7.4)	
Sleep quality			
Good (<3)	18.4 (14.0, 22.7)	17.6 (14.8, 20.4)	NS
Fair (≥3 to <7)	23.8 (19.8, 27.8)	21.3 (17.8, 24.7)	
Poor (≥7 to <12)	29.9 (24.2, 35.5)	27.3 (23.4, 31.2)	
Very poor (≥12 to 24)	28.0 (23.7, 32.2)	33.8 (30.4, 37.2)	
Physician diagnosed sleep disorder			
No	90.1 (86.3, 94.0)	83.8 (80.7, 86.8)	<0.05
Yes	9.9 (6.0, 13.7)	16.2 (13.2, 19.3)	

Mean (95% CI) for continuous variables and % (95% CI) for categorical variables. MHO is metabolically healthy obesity, based on the Joint Interim Statement of metabolic syndrome. *p* < 0.05, two-sided; independent *t*-test or *χ*^2^, as appropriate. NS is not significant.
